# Contribution of Immune Cells to Glucocorticoid Receptor Expression in Breast Cancer

**DOI:** 10.3390/ijms21134635

**Published:** 2020-06-30

**Authors:** Shipra Gandhi, Ahmed Elkhanany, Masanori Oshi, Tao Dai, Mateusz Opyrchal, Hemn Mohammadpour, Elizabeth A. Repasky, Kazuaki Takabe

**Affiliations:** 1 Department of Medical Oncology, Roswell Park Comprehensive Cancer Center, Elm & Carlton Streets, Buffalo, NY 14263, USA; 2 Department of Medical Oncology, University of Alabama, Birmingham, AL 35294, USA; aelkhanany@uabmc.edu; 3 Breast Surgery, Department of Surgical Oncology, Roswell Park Comprehensive Cancer Center, Buffalo, NY 14263, USA; masanori.oshi@roswellpark.org (M.O.); Kazuaki.Takabe@roswellpark.org (K.T.); 4 Departments of Surgery, Yokohama City University, Yokohama 236-0004, Japan; 5 Department of Immunology, Roswell Park Comprehensive Cancer Center, Buffalo, NY 14263, USA; tao.dai@roswellpark.org (T.D.); hemn.mohammadpour@roswellpark.org (H.M.); Elizabeth.Repasky@RoswellPark.org (E.A.R.); 6 Division of Medical Oncology, Washington University, St. Louis, MO 63130, USA; m.opyrchal@wustl.edu; 7 Department of Surgery, Niigata University Graduate School of Medical and Dental Sciences, Niigata 951-8510, Japan; 8 Department of Breast Surgery and Oncology, Tokyo Medical University, Tokyo 160-8402, Japan; 9 Department of Breast Surgery, Fukushima Medical University, Fukushima 960-1295, Japan

**Keywords:** glucocorticoid receptor, breast cancer, *NR3C1*, immune cells, TCGA, METABRIC, CIBERSORT

## Abstract

Breast cancer (BC) patients experience increased stress with elevated cortisol levels, increasing risk of cancer recurrence. Cortisol binds to a cytoplasmic receptor, glucocorticoid receptor (GR) encoded by GR gene (*NR3C1*). We hypothesized that not only cancer cells, but even immune cells in the tumor microenvironment (TME) may contribute to GR expression in bulk tumor and influence prognosis. To test this, mRNA expression data was accessed from METABRIC and TCGA. “High” and “low” expression was based on highest and lowest quartiles of *NR3C1* gene expression, respectively. Single-cell sequencing data were obtained from GSE75688 and GSE114725 cohorts. Computer algorithms CIBERSORT, Gene Set Enrichment Analysis and TIMER were used. GR-high BC has better median disease-free and disease-specific survival. Single cell sequencing data showed higher GR expression on immune cells compared to cancer and stromal cells. Positive correlation between GR-high BC and CD8^+^ T-cells was noted. In GR-high tumors, higher cytolytic activity (CYT) with decreased T-regulatory and T-follicular helper cells was observed. High GR expression was associated with lower proliferation index Ki67, enriched in IL-2_STAT5, apoptosis, KRAS, TGF-β signaling, and epithelial-to-mesenchymal transition. Immune cells significantly contribute to GR expression of bulk BC. GR-high BC has a favorable TME with higher CYT with favorable outcomes.

## 1. Introduction

Although often ignored and under-appreciated, breast cancer patients suffer from potentially debilitating stress, anxiety, depression, and impaired cognitive function [1,2]. Stress has been demonstrated to result in an increased incidence of cancer recurrence [3]. On the contrary, breast cancer patients with no stressful or traumatic life events have significantly longer disease-free intervals compared to patients who have experienced these events [3].

Stress activates the hypothalamic–pituitary–adrenal axis with corticotropin-releasing factor produced in the hypothalamus, which stimulates the release of adrenocorticotrophic hormone (ACTH) from the anterior pituitary. ACTH signals the adrenal cortex to produce glucocorticoids—the stress hormone ‘cortisol’ secreted by the zona fasciculata of the adrenal glands. Cortisol levels are elevated in patients with breast cancer and the diurnal cortisol rhythm is a predictor of breast cancer survival [4]. Cortisol generates physical response to stress by binding to its cytoplasmic receptor, glucocorticoid receptor (GR), which is the transcription factor encoded by the *NR3C1* gene [5], thus promoting “stress response” [6,7].

The role of GR activation has been reported to be different between breast cancer subtypes, namely, estrogen receptor (ER)-negative and ER-positive breast cancer. Activation of GR in ER-negative human breast cancer cell lines has been shown to promote cancer cell survival, chemotherapy resistance, and increased tumor growth in a pre-clinical xenograft model [8,9]. A retrospective meta-analysis in primary breast tumors showed that high gene expression of GR (*NR3C1*) in the bulk tumor was associated with significantly worse relapse-free survival (RFS) among ER-negative early stage breast cancer, but better survival in ER-positive breast cancer. It was speculated that this difference is due to an interaction of the GR and ER [10].

The levels of stress hormones, cortisol and corticosterone, were higher in the plasma of mice with metastatic breast cancer than in healthy controls. Obradovic et al. showed that increase in stress hormones during breast cancer progression results in activation of GR at distant metastatic sites, increased colonization and reduced survival [11]. Studies have shown that strategies to antagonize GR signaling can sensitize ovarian, prostate and triple negative breast cancer (TNBC) cell lines to chemotherapy. Therefore, inhibition of the GR pathway is being investigated in clinical trials combining GR/PR antagonist mifepristone with nab-paclitaxel [12].

Although the role of GR activation in chemoresistance and enhanced aggressive phenotype have been studied both in vitro [13] and in vivo [13], both models lack immune cells. It is well known that TNBC have more immune cell infiltration than ER-positive tumors [14]. Immune cells, including T-cells, B-cells, monocytes, neutrophils, and macrophages, also express the GR, in addition to cancer cells in the bulk tumor [15]. In addition to the downstream effects of GR activation on tumor cells, over the last few years, there has been interest in understanding GR expression on immune cells in the bulk tumor [16] and the impact of its activation [17,18].

Recent computational biological analyses of transcriptomic data of bulk tumors allow us to investigate human tumor immune microenvironment (TME) in large cohorts. Here, we hypothesized that not only cancer cells, but immune cells in the TME also contribute to the GR expression of the bulk tumor, which may contribute to differences in outcome.

## 2. Results

### 2.1. Demographic and Clinical Characteristics

There were 1390 and 1022 patients with stage I–III breast cancer with clinical and genomic data available in Molecular Taxonomy of Breast Cancer International Consortium (METABRIC) and The Cancer Genome Atlas (TCGA), respectively. High and low tumor GR expression was defined as the highest and lowest quartile (25%) of *NR3C1* expression, respectively (Figure S1). Table 1 shows the distribution of demographic (age at diagnosis) and clinical characteristics (stage at diagnosis, clinical subtypes, PAM50 subtypes) among GR-high and GR-low breast cancer based on GR expression in METABRIC and TCGA. There were 696 (METABRIC) and 512 patients (TCGA) in the top and bottom quartiles from the entire cohort of 1390 and 1022 patients, respectively. No statistically significant distribution in age at diagnosis was observed. Clinical subtypes were equally distributed between GR-high vs. GR-low cohorts except HER2-positive subtype, which was higher in GR low cohort in METABRIC. There were more patients with hormone receptor positive subtype in GR-high vs. more triple negative subtype (65.1%) in GR-low cohorts in TCGA. There were more stage 1 patients observed in GR high cohort vs. more stage 2 patients in GR low cohort. Hence, there was a difference in stage distribution noted. Differences in clinical subtypes were observed in both cohorts and hence, subgroup analyses for receptor subtypes has been performed to understand the influence of GR expression in breast cancer subtypes. Both METABRIC and TCGA datasets are limited by the unavailability of treatment information.

### 2.2. GR-high Breast Cancer has Better Survival

**Figure 1:**
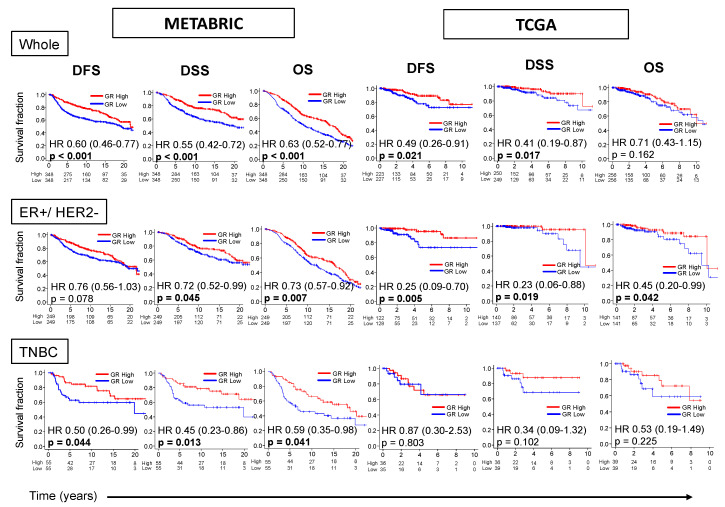
GR expression and survival outcomes in breast cancer. Kaplan–Meier survival plots comparing patients with high and low GR expression along with logrank test *p* values and hazard ratios (HR) with confidence intervals are shown for disease-free (DFS), disease-specific (DSS) and overall survival (OS) for the entire cohort (Whole), or its sub-groups of estrogen receptor(ER)-positive/human epidermal growth factor receptor (HER2)-negative and triple negative (TNBC) breast cancer. The cut-off of top and bottom quartile of *NR3C1* expression was considered as GR high and GR low in the whole cohort and the respective subtypes. Log-rank test was used to compare the survival between GR high and GR low breast cancer.

Survival characteristics of GR expression in METABRIC and TCGA cohorts are shown in Figure 1. GR-high tumors have better median disease-free survival (mDFS) 21.7 vs. 19.3yrs {HR 0.60 (0.46–0.77), *p* < 0.001} and better median disease-specific survival (mDSS) NR vs. 19.9yrs {HR 0.55 (0.42–0.72), *p* < 0.001} in METABRIC. This survival difference was consistent in TCGA cohort, but more significant in the METABRIC cohort, which had a larger cohort size. GR-high tumors were also noted to have better median overall survival (mOS) 16.6 vs. 10.1yrs {HR 0.63 (0.52–0.77), *p* < 0.001} in METABRIC.

Due to a previously reported notion on the interaction between GR and ER, and different survival of GR-high vs. GR-low groups in ER-positive and TNBC subtypes as described previously [10], the influence of receptor subtype on survival characteristics was also analyzed. Estrogen receptor (ER)-positive/human epidermal growth factor receptor (HER2)-negative subtype had better mDSS (*p* < 0.05) and mOS (*p* < 0.05) in GR-high compared with GR-low tumors in both cohorts. TNBC subtype was associated with better mDFS {HR 0.50 (0.26–0.99), *p* = 0.044}, mDSS {HR 0.45 (0.23–0.86), *p* = 0.013} and mOS {HR 0.59 (0.35–0.98), *p* = 0.041} in GR-high compared with GR-low tumors in METABRIC, but only a trend towards better survival without statistical significance in TCGA (Figure 1). Survival difference between GR-high vs. GR-low breast cancer for the entire ER-positive breast cancer and HER2-positive breast cancer subtypes has been shown in Figure S2. Among ER-positive subtype, GR-high breast cancer was associated only with better mDFS validated in TCGA as has been shown before. Among HER-2 positive subtype, GR-high breast cancer was associated with better survival in METABRIC, but not validated in TCGA. Thus, our data shows that GR expression in the bulk tumor is associated with an improvement in survival, mostly in ER-positive/HER2-negative breast cancer subtype.

### 2.3. Immune Cells have High GR Expression than Tumor and Stromal Cells

**Figure 2:**
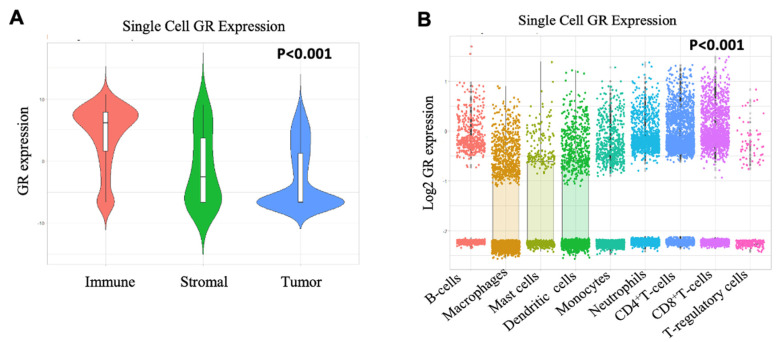
Single cell sequencing data of primary breast cancer. (**A**) GSE75688 shows that immune cells have higher GR expression than tumor and stromal cells. Data compiled from GSE cited was used to perform one-way ANOVA across all cell subsets. The *p* value is for the F statistic within ANOVA. (**B**) GSE114725 shows that CD8^+^ T-cells have higher GR expression than other immune cell subsets. Data compiled from GSE cited was used to perform one-way ANOVA across immune cell subsets. The *p* value is for the F statistic within ANOVA. Bonferroni correction was used to compare CD8^+^ T-cells to other immune cell subsets with higher average mean in post-hoc analysis.

Single cell sequencing technology provides a higher resolution of the cellular differences and a better understanding of the function of an individual cell [19]. Since published literature shows differences in survival analyses of GR-high vs. GR-low groups between ER-positive and TNBC subtypes [10], hence, we hypothesized that there could be some contribution of non-tumor cells to GR expression. Therefore, single cell sequencing dataset was used to analyze GR expression differences between immune and tumor cells. Interestingly, higher GR expression was observed on immune cells (T-cells, B-cells and myeloid cells) compared to stromal or cancer cells (*p* < 0.001) (GSE75688) (Figure 2A), with the highest GR expression on CD8^+^ T-cells compared to other immune cell subsets including regulatory T-cells (T-regs), CD4^+^ T-cells, neutrophils, monocytes, dendritic cells, mast cells, and macrophages (*p* < 0.001) (GSE114725) (Figure 2B).

### 2.4. CD8^+^ T-cells Significantly Correlate with GR Expression

**Figure 3:**
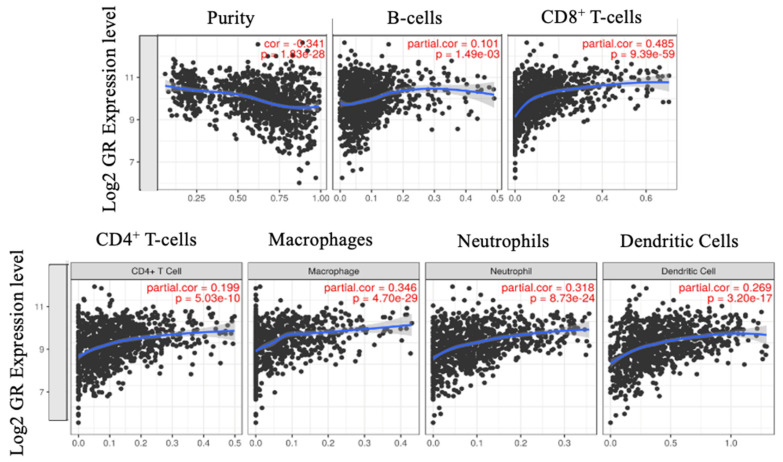
Spearman rho correlation was used to assess correlation between GR expression and immune cells. Correlation is quantified as weak (absolute value < 0.3), moderate (absolute value between 0.3 and 0.5) and strong (absolute value > 0.5) in either direction. Moderate correlation observed between CD8^+^ T-cells and macrophages and GR expression in breast cancer using TIMER. This moderate positive correlation between GR expression and macrophages was not, however, validated using other deconvolution algorithms.

We observed that immune cells contribute to GR expression, hence, it was of interest to investigate if immune cell markers correlated with GR expression in the bulk tumor. We ran TCGA data through TIMER to quantify immune cell composition across GR gene expression. There was significant correlation between GR (Log-value) and absolute CD8^+^ T-cell fraction (as calculated by TIMER) as well as absolute macrophages and GR expression (Spearman r = 0.485 and 0.346 respectively; *p* < 0.01). There were weak correlations between GR expression and B-Cells, dendritic cells, CD4^+^ T-cells, and neutrophils (Figure 3). Since we observed minimal GR expression on macrophages but a moderate positive correlation between GR expression and macrophages, we analyzed the correlation between GR expression and macrophages across different immune cell composition algorithms as shown in Figure S3. No correlation between GR expression and macrophages was observed with the other algorithms. TIMER is designed to analyze exclusively RNA-sequence data, thus METABRIC cohort, which utilized gene expression microarray, was not analyzed.

### 2.5. GR-high Breast Cancer has Higher Cytolytic Activity

**Figure 4:**
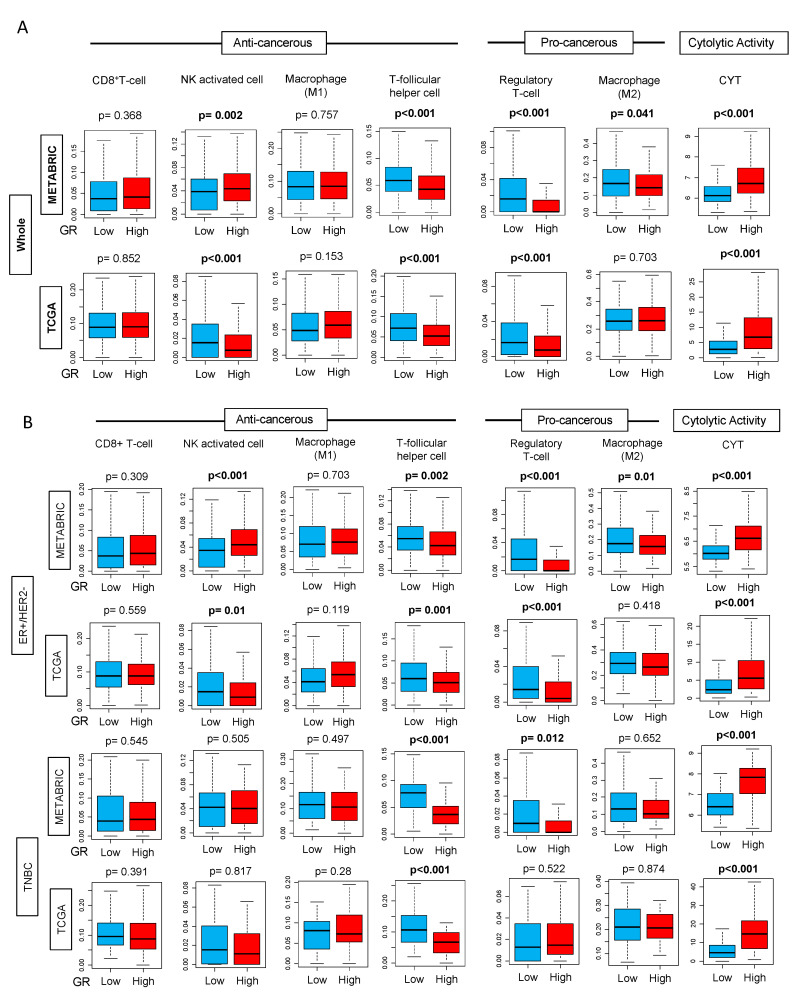
Tukey boxplots of immune cells CD8^+^ T-cells, Natural Killer (NK) activated cells, M1 and M2 macrophages, T-follicular helper cells, T-regulatory cells, and cytolytic activity (CYT) in GR-high and GR-low breast cancer using CIBERSORT algorithm in METABRIC and TCGA cohorts (**A**) in the entire cohort (Whole) and (**B**) in estrogen receptor (ER)-positive/human epidermal growth factor receptor (HER2)-negative and triple negative (TNBC) subtypes. The cut-off of top and bottom quartile of NR3C1 expression was considered as GR high and GR low in the entire cohort and also in the respective subtypes. *Y*-axis shows the fraction of cells with GR-low or GR-high expression. Boxes depict medians and interquartile ranges. Depicted *p* values are calculated using one-way ANOVA.

Since we observed higher GR expression on the immune cells compared to stromal and tumor cells in single cell sequencing dataset and positive correlation of CD8^+^ T-cells with GR expression, we investigated the relative distribution of individual immune cell subpopulation in bulk cancer between GR-high vs. GR-low groups in METABRIC using CIBERSORT algorithm. Immune cell subpopulations, including anti- and pro-cancerous immunosuppressive cells, were compared between the two groups. GR-high tumors had a significantly lower number of immunosuppressive T-regs (*p* < 0.001) (Figure 4A) but at the same time, GR-high cohort also had a lower number of anti-cancerous T-follicular helper cells (*p* < 0.001). In addition, we also examined the differences in cytolytic activity (CYT) defined as the expression of granzyme A (GZMA) and perforin (PRF1) as described in Materials and Methods. Overall, GR-high cohort had higher CYT (*p* < 0.001), which could explain the higher DFS and DSS observed in this group (Figure 4A). Strikingly consistent results were seen within the TCGA cohort. Due to known differences in survival between GR-high vs. GR-low breast cancer in ER-positive and TNBC subtypes, we analyzed the contribution of immune cells and CYT in these subtypes. Figure 4B shows the distribution of these immune cells between GR-high and GR-low groups in ER-positive/HER2-negative breast cancer and TNBC subtypes. We observed similar findings with the lower number of T-follicular helper cells (*p* < 0.01) in the GR-high group in both METABRIC and TCGA in the two subtypes. In ER-positive/HER2-negative subtype, lower T-regs were observed in GR-high breast cancer (*p* < 0.001); this finding was validated in TNBC in METABRIC, but not in TCGA. The subtype analyses further validated that there is higher CYT in the GR-high group.

### 2.6. GR-high Breast Cancer has More T-cell Exhaustion Markers

**Figure 5:**
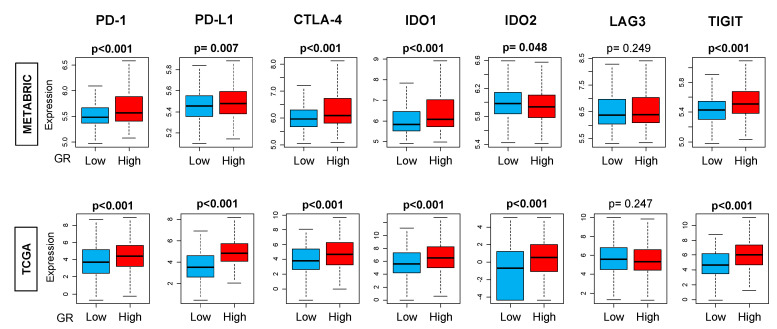
Immune exhaustion gene markers in GR-high and GR-low groups using *CIBERSORT* algorithm in the entire cohort. The cut-off of top and bottom quartile of *NR3C1* expression was considered as GR high and GR low in the entire cohort. *Y*-axis shows the fraction of cells with GR-low or GR-high expression. All boxplots are of Tukey type, and the boxes depict medians and interquartile ranges. Depicted *p* values are calculated using one-way ANOVA. PD-1, programmed death-1; PD-L1, programmed death ligand 1/2; CTLA-4, cytotoxic T-lymphocyte-associated protein 4; IDO, indoleamine 2,3-dioxygenase; LAG3, lymphocyte activation gene 3; TIGIT, T cell immunoreceptor with Ig and ITIM domains.

Due to differences in immune cells and CYT between GR-high and GR-low breast cancer, we explored the distribution of immune exhaustion gene expression markers. Based on the results from CIBERSORT and TIMER, we hypothesized that T-cell exhaustion markers should be elevated in GR-high tumors due to the higher presence of immune cells. T-cell exhaustion gene expressions were analyzed in GR-high vs. GR-low tumors. We observed a significantly higher expression of T-cell exhaustion genes PD-1, PD-L1, CTLA-4, IDO1, and TIGIT in GR-high tumors (*p* < 0.01) (Figure 5).

### 2.7. GR-high Breast Cancer is Enriched in IL2 STAT5, Apoptosis, KRAS, TGF-β, EMT Pathways and GR-low Breast Cancer has Higher Proliferation Markers

**Figure 6:**
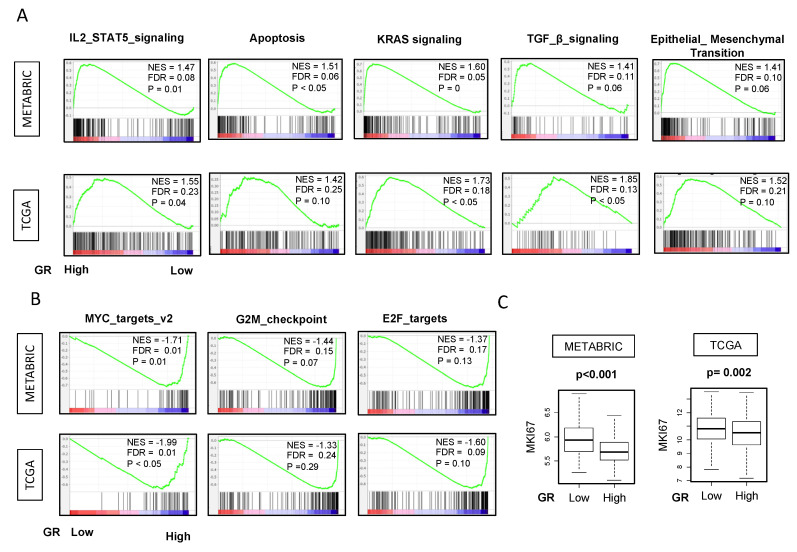
Hallmark gene sets with significant enrichment in GR high and low breast cancer in both METABRIC and TCGA cohorts. Gene set enrichment (GSEA) plots along with normalized enrichment score (NES) and false discovery rate (FDR) are shown for the gene sets for which enrichment was seen in (**A**) GR high and (**B**) GR low tumors in both METABRIC and TCGA in the entire cohort. NES and FDR were determined with the classical GSEA method. The cut-off of highest and lowest quartiles (25%) of NR3C1 expression was considered as GR high and GR low. The statistical significance of GSEA was determined using FDR of 0.25. Nominal *p*-value estimates the significance of the observed enrichment score for a single gene set. However, FDR is the estimated probability that a gene set within a given enrichment score (normalized for gene set size) represents a false positive finding as recommended by Broad Institute. (**C**) GR low tumors were associated with high MKI67 transcriptome analysis in both METABRIC and TCGA in the entire cohort. Depicted *p* values are calculated using one-way ANOVA.

Given our findings, we hypothesized that immune-related gene sets would be enriched in GR-high tumors. In order to examine the different immunological pathways to explain the disparities, we ran GSEA on METABRIC and TCGA cohorts. Most notably, IL-2_STAT5 and apoptosis pathways were enriched in GR-high compared to GR-low breast cancer explaining higher inflammatory response and better survival. However, to our surprise, enrichment of TGF-β, KRAS and epithelial-to-mesenchymal transition (EMT) pathways were also noted in the same group as well which are associated with worse prognosis and relative immune-resistance (other clinically insignificant pathways which are enriched include UV response, and complement pathways) (FDR < 0.25) (Figure 6A).

We also hypothesized that GR-low breast cancer would be associated with more proliferation and worse outcomes due to lower anti-tumor response with lower CYT. Strikingly, we observed that GR-low breast cancer was significantly enriched in gene sets related to cell proliferation MYC_TARGETS_v2. Furthermore, GR-low breast cancer was also significantly enriched in gene sets related to cell cycle, such as E2F TARGETS and G2M_CHECKPOINT (Figure 6B).

This notion was further confirmed by the transcriptome analysis of Ki67, which is one of the most commonly used markers for cell proliferation. GR-high breast cancers were associated with significantly lower Ki67 expression (Figure 6C). Hence, GR-high tumors are enriched in gene sets for immune response and apoptosis with lower proliferation consistent with better survival. Figure S4 shows all the hallmark gene sets with significant enrichment in GR high and GR low breast cancer with FDR < 0.25.

## 3. Discussion

In this study, we examined the tumor microenvironment differences between GR-high and GR-low breast cancer and explored role of immune cells in GR expression of the bulk tumor to explain the disparities in outcomes in the two cohorts. This is in addition to the well-known direct transcriptional role of GR on tumor cells which plays a critical role in clinical outcomes.

Our study shows that GR-high breast cancer has better survival compared to GR-low breast cancer, particularly in ER-positive/HER2-negative breast cancer. This is consistent with previous retrospective studies showing that GR expression in ER-positive breast cancer is associated with better outcomes [10,13,20]. On the contrary, previous studies have shown that GR expression in ER-negative breast cancer was associated with worse RFS. Our analysis showed that high GR expression was associated with better outcomes in TNBC in METABRIC but not validated in TCGA where only a trend towards improved survival was seen with no statistical significance. Our findings in TNBC subtype did not replicate results from previous publications probably due to the different data cohort (TCGA and METABRIC) and methodology for ER-positivity used here [10,13,20]. As published by Conzen et al. in *Cancer Research*, 8 Gene Expression Omnibus (GEO) studies were combined and expression of ESR1 was used to categorize samples into specific subtypes with 1378 patients (1024 ESR1 positive and 354 ESR1 negative) [10]. A combination of studies is less ideal than using a single cohort that has been curated and standardized in uniform manner, because of different sequencing techniques used (variable standardization). In addition to these differences, we investigated the contribution of immune cells to explain this discrepancy in outcomes.

It is well known in literature that the presence of T-regs is associated with poor RFS and worse outcomes in breast cancer [21]. To our knowledge, this is the first study to find significant differences in the immune cell landscape in the GR-high vs. GR-low breast cancer. In our current study, we observed that there are a lesser number of immunosuppressive T-regs in GR-high tumors. On the other hand, we also found lower anti-tumor T-follicular helper cells [22] in the GR high tumors. Although there are both lower immunosuppressive and anti-tumor cells, overall we observed a higher cytolytic activity in GR-high tumors. It is interesting to note that although we observed enrichment of TGFβ signaling in GR-high breast cancer, there were lower Tregs in this group. Prior studies have shown that in addition to TGFβ, other cytokines such as IL-10, IL-4 and IL-13 are also involved in Treg generation and induction, and it is possible that these other cytokines could be implicated in regulating Tregs in our study [23]. In addition, we found that GR-high breast cancers have a higher immune response with higher IL-2 pathway and apoptosis and lower proliferation, explaining improved survival. However, this is in addition to higher TGF-β score, KRAS and EMT pathways. TGF-β may promote or inhibit tumor progression [24,25], whereas KRAS and EMT pathways are associated with worse prognosis, though there is still a lack of data for the role of KRAS in breast cancer [26,27]. It is interesting to note that while KRAS and EMT pathways that result in aggressive tumor biology are enriched in the GR-high group, immune pathways IL2 and apoptosis are also enriched, which may play a role in influencing overall survival. The importance of anti-tumor immune response in influencing overall prognosis and outcomes in breast cancer is evident from literature [28]. Prior studies have also shown a similar GR-associated modulation of immune response pathway genes [10] in ER-negative subtype, but we observed a difference in TME both in ER+/HER2 negative and TNBC subtypes. As is published in the literature, in addition to a direct transcriptional role of GR on the tumor cells, which could be mediated by EMT pathways elevated in the GR-high group, the contribution of immune cells to outcomes should also be acknowledged because of a potential interaction of GR activity with the TME.

Breast cancer patients suffering from stress have shorter survival compared to patients who do not report stress [3]. Stress mediated cortisol release in peripheral blood acts on the cytoplasmic GR resulting in cancer cell survival, chemotherapy resistance, and increased tumor growth in pre-clinical models as well as plays a role in regulation of immune system [8,11,29,30]. Studies have investigated the variation in the expression of GR on immune cells [15] with stress and have shown different findings. Even among the immune cells, the relative expression of GR varies among different cells, with higher expression in eosinophils, followed by granulocytes, T lymphocytes and NK cells (*p* < 0.05) [31], however, no correlation was observed between serum cortisol and GR expression on the leucocyte subpopulations [31]. On the other hand, another study showed reduced peripheral expression of the GRα isoform on the peripheral blood cells in individuals with post-traumatic stress disorder: a cumulative effect of trauma burden [32]. Although our study showed that CD8^+^ T cells have higher GR expression, however, we did not see any difference in their distribution between GR-high and GR-low breast cancer. Similarly, although macrophages have minimal GR expression, a moderate positive correlation of GR expression with macrophages was observed using TIMER, however, this correlation was not validated in more robust deconvolution algorithms. At the same time, we did not see any difference in macrophage distribution between GR-high and GR-low breast cancer. This leads us to hypothesize that immune cells in addition to macrophages and CD8^+^ T-cells are likely to be contributing to the GR signature of the bulk tumor, though none of the immune cells were independently elevated in GR-high breast cancer. It is well known that GR signaling influences functions of different immune cells. Glucocorticoids exert anti-inflammatory activity by inhibiting neutrophil rolling, adhesion and activation; they inhibit dendritic cells to activate T-cells; favor T-cell apoptosis by acting on T-helper 1 (Th1) cell by decreasing T-bet transcriptional activity and suppressing the production of pro-inflammatory molecules IL-2 and IFN-γ that favors T-reg expansion [33].

Single-cell RNA sequencing provides a new platform to understand the dynamic ecosystem that comprises of tumor cells, fibroblasts and immune cells. Gene expression data from bulk tumors is indispensable and continues to dominate the clinical and translational settings, however, TCGA designs have been focused on the cancer cells (high amount of cancer cells were one of the criteria of the sampling of the tissue), whereas single-cell RNA sequencing data can capture the gene expressions of cells in the surrounding stroma such as immune cells [34,35]. Single-cell sequencing technologies hold the potential to revolutionize the field of cancer [36]. We pursued analysis of single cell sequencing data in order to analyze the contribution of GR expression on the immune cells (contributing to the bulk genomic and transcriptomic signature). Interestingly, our single-cell sequencing data show that immune cells express significantly higher GR compared to other tumor and stromal cells, and thus, contribute to the GR expression of the bulk tumor.

Since immune cells also express GR, we speculate that modulation of GR signaling in the presence of cortisol may cause their activation/suppression, which may additionally contribute to the different outcomes in GR-high versus GR-low groups. Our observation that there are lesser number of immunosuppressive pro-cancerous T-regs in GR-high tumors may consolidate the findings from a previous study [32] as patients with less stress (less cortisol) may have higher GR expression with lower number of T-regs, thus higher CYT and hence, improved survival. This provides a hypothesis that in addition to a direct transcriptional role of GR on tumor cells and interaction with ER, there may be an additional role of immune cells in GR-high vs. GR-low tumors in influencing prognosis and potentially as a therapeutic strategy in addition to targeting the GR expression on tumor cells. Our hypothesis is further strengthened by the recent approval of checkpoint inhibitors in breast cancer with high PD-L1 expression on immune cells [37], further highlighting that immune-mediated pathways are crucial and present an excellent opportunity for targeted approaches to overcome underlying immunosuppression in breast cancer and improve outcomes.

Our study limitations include analysis from a publicly available database and also limited data interpretation by lack of a mechanistic approach and causality association as this study does not contain in vitro and in vivo data. The finding of different immune cell subpopulations in GR-high and GR-low groups and the contribution to outcomes is hypothesis-generating and needs mechanistic validation. Future work needed to advance this field further should focus on investigating if GR expression correlates with GR signaling by analyzing downstream pathways in different subtypes of breast cancer, especially receptor-tyrosine-kinase-like orphan receptor 1 (ROR1) signaling, which has been shown to be associated with aggressive disease and decreased survival in breast cancer [11,38]. In addition, this would also help elucidate if higher GR expression on immune cells corresponds to increased or decreased sensitivity towards GR signaling/activation and immunosuppression.

## 4. Materials and Methods

### 4.1. Obtaining Data of METABRIC and TCGA

Molecular Taxonomy of Breast Cancer International Consortium (METABRIC) dataset [39] was accessed from cBioPortal [40,41,42,43,44]. Annotated clinical and outcome data as well as gene expression data for 1903 breast cancer patients were downloaded and analyzed as we described previously [45,46]. The Cancer Genome Atlas (TCGA) was also accessed from cBioPortal [47]. RNA-Sequencing data of 1093 breast cancer patients were downloaded. Of these, we selected patients with American Joint Committee on Cancer (AJCC) staging I, II and III, and used them for the study. Clinical data, outcome data and immune composition data were downloaded from PANCAN publications and outcome measures reported as we have described previously [46,48,49,50,51,52,53,54,55,56]. Disease-free survival (DFS) was defined from the time of completion of primary treatment until clinical confirmation of tumor recurrence. Overall survival (OS) was defined as the time from treatment completion until death. Disease-specific survival (DSS) was defined as the time from treatment completion until death, however, patients who die of causes other than the disease were excluded. We classified the patients into two groups based on GR expression. Highest and lowest quartile (25%) of *NR3C1* expression was used as a cutoff to identify “high” and “low” GR (*NR3C1*) tumor expression, respectively. This study was deemed exempt from Institutional Review Board because all information within TCGA and METABRIC is publicly accessible and de-identified [50,54,55,57].

### 4.2. Immune Analysis Using CIBEROSRT Algorithm

Immune composition data were downloaded from PANCAN Immune landscape project [58]. The project used CIBERSORT, a bioinformatics algorithm [59], to predict immune composition among METABRIC and TCGA samples, utilizing a set of 22 immune cell reference profiles and developing a signature matrix to predict their absolute levels within each sample, as we described previously [45,46,47,60,61,62]. Cytolytic activity ‘CYT’ is defined as an algorithm calculated using the expression of granzyme A (GZMA) and perforin (PRF1) by Rooney et al. published in Cell 2015 [63]. Data for CYT has been described in previous work and appended into the analysis [47,61,63,64,65,66,67]. This is a simple and quantitative measure of immune cytolytic activity ‘CYT’ based on the transcript levels of two key cytolytic activity-related genes, GZMA and PRF1, which have been observed to be dramatically upregulated upon CD8^+^ T-cell activation. Tumor IMmune Estimation Resource (TIMER) online portal was accessed for independent validation of results [68].

### 4.3. Gene Set Expression Analysis

Publicly available software provided by the Broad Institute was used to perform gene set enrichment analysis (GSEA) [69] as we have described previously [46,48,50,54,70,71,72,73,74]. In GSEA, the nominal *p* value estimates the significance of the observed enrichment score for a single gene set. For evaluation of multiple gene sets, false discovery rate (FDR) is used to correct for multiple hypothesis testing. The FDR is the estimated probability that a gene set within a given enrichment score (normalized for gene set size) represents a false positive finding. As recommended by the Broad Institute (that developed GSEA), FDR of less than 0.25 was used to define statistical significance of GSEA. An FDR of 25% indicated that the result is likely to be valid three out of four times, which is reasonable in the setting of exploratory discovery where one is interested in finding a candidate hypothesis to be further validated as a result of future research. Given the lack of coherence in most expression datasets and the relatively small number of gene sets being analyzed, using a more stringent FDR cutoff could lead us to overlook potentially significant results. Hallmark gene sets were used for this study.

### 4.4. Statistical Analysis

Clinical characteristics between groups were analyzed by χ squared distribution (for categorical variables) and student T-test, Wilcox rank sum and Kruskal Wallis (for continuous variables). Survival statistics were obtained using Kaplan–Meier method with log-rank test. Cumulative incidence of recurrence was calculated based on DFS, with death handled as a competing risk event. CIBERSORT immune cell composition was compared between the two cohorts via one-way ANOVA. Single cell sequencing data were obtained from primary breast cancer GSE75688 and GSE114725 datasets. All statistical analyses were performed using STATA software (version 15.1; STATA, College Station, TX), R software (version 3.6.2). In all analysis, a two-sided *p* < 0.05 was considered as statistically significant. All boxplots are of Tukey type, and the boxes depict medians and inter-quartile ranges.

## 5. Conclusions

Our study shows that GR-high tumors have favorable outcomes, mostly in ER positive breast cancer subtype, which is consistent with previous results. Immune cells significantly contribute to GR expression of the bulk breast tumor in addition to tumor cells. GR expression correlated with higher CD8^+^ T-cells. GR-high tumors have a favorable tumor microenvironment with higher cytolytic activity. Additional work exploring the relative contribution and factors influencing the activation/inactivation of these immune cells with GR signaling in the tumor microenvironment is warranted.

## Figures and Tables

**Table 1:** Demographic and clinical characteristics of the GR High and GR low breast cancer patients in METABRIC and TCGA in the entire cohort.

Demographic and Clinical Characteristics	METABRIC			TCGA		
Clinical Variables (percent per GR status)	GR-LOW *n* = 348	GR-HIGH *n* = 348	*p*-value	GR-LOW *n* = 256	GR-HIGH *n* = 256	*p*-value
Age at diagnosis			0.063			0.376
Median	63	61		59	60	
IQR	53–71	52–69		50–69	50–66	
Stage at diagnosis			0.017			0.019
I	104 (29.9%)	140 (40.2%)		36 (14.0%)	56 (21.9%)	
II	215 (61.8%)	182 (52.3%)		165 (64.5%)	135 (52.7%)	
III	29 (8.3%)	26 (7.5%)		55 (21.5%)	65 (25.4%)	
Clinical Subtypes						
ER Positive	257 (48.1%)	277 (51.9%)	0.088	183 (47.2%)	205 (52.8%)	0.020
PR Positive	188 (51.8%)	175 (48.2%)	0.363	152 (45%)	186 (55%)	0.001
HER2 Positive	59 (71.1%)	24 (28.9%)	<0.001	54 (65.1%)	29 (34.9%)	<0.001
Triple Negative	55 (49.1%)	57 (50.9%)	0.918	28 (65.1%)	15 (34.9%)	0.033
PAM50 Subtypes			<0.001			<0.001
Luminal A	117 (33.6%)	137 (39.4%)		65 (25.4%)	149 (58.2%)	
Luminal B	102 (29.3%)	64 (18.4%)		45 (17.6%)	34 (13.3%)	
HER2	59 (17%)	24 (6.9%)		21 (8.2%)	7 (2.7%)	
Basal	57 (16.4%)	47 (13.5%)		30 (11.7%)	27 (10.5%)	

Differences between GR-low and GR-high groups were tested for statistical significance using Fisher’s exact test. IQR = Interquartile range, HER2 = human epidermal growth factor receptor2.

## Supplementary Materials

Supplementary materials can be found at https://www.mdpi.com/1422-0067/21/13/4635/s1. Figure S1: Highest and lowest quartiles (25%) of *NR3C1* RNA-seq expression as a cutoff to identify “High” and “Low” GR (*NR3C1*) tumor expression, respectively, Figure S2: GR expression and survival outcomes in breast cancer. Kaplan-Meier survival plots comparing patients with high and low GR expression along with logrank test p values and hazard ratios (HR) with confidence intervals are shown for disease-free (*DFS*), disease-specific (*DSS*) and overall survival (*OS*) for sub-groups of (A) estrogen receptor (*ER*)-positive and (B) human epidermal growth factor receptor (*HER2*)-positive breast cancer. The cut-off of top and bottom quartile of *NR3C1* expression was considered as GR high and GR low in the respective subtypes. Log-rank test was used to compare the survival between GR high and GR low breast cancer, Figure S3: Correlation between GR expression and macrophages across different immune cell composition algorithms (EPIC, TIMER, CIBERSORT, xCELL), Figure S4: Hallmark gene sets with significant enrichment in GR high and low breast cancer with FDR < 0.25.

## Abbreviations

ACTH	Adrenocorticotrophic hormone
DSS	Disease-specific survival
CYT	Cytolytic activity
DFS	Disease-free survival
ER	Estrogen receptor
EMT	Epithelial-to-mesenchymal transition
GR	Glucocorticoid receptor
GSEA	Gene set enrichment analysis
METABRIC	Molecular Taxonomy of Breast Cancer International Consortium
NK	Natural killer
OS	Overall survival
RFS	Relapse-free survival
TCGA	The Cancer Genome Atlas
TGF-β	Transforming growth factor-beta
TME	Tumor immune microenvironment
TNBC	Triple negative breast cancer
T-regs	Regulatory T-cells
GEO	Gene Expression Omnibus
