# Supplementary material for: Contribution of Immune Cells to Glucocorticoid Receptor Expression in Breast Cancer
Source: Int J Mol Sci. 2020 Jun 30;21(13):4635. doi: 10.3390/ijms21134635 (PMC7370149; doi:10.3390/ijms21134635)
Supplement: Supplementary file 1 [file ijms-21-04635-s001.pdf]

SUPPLEMENTARY MATERIALS

Gandhi et al. "**Contribution of immune cells to glucocorticoid receptor expression in breast cancer.**"

Content

1. Figure S1: Highest and lowest quartiles (25%) of *NR3C1* RNA-seq expression as a cutoff to identify "High" and "Low" GR (*NR3C1*) tumor expression, respectively.

2. Figure S2: GR expression and survival outcomes in breast cancer. Kaplan-Meier survival plots comparing patients with high and low GR expression along with logrank test p values and hazard ratios (HR) with confidence intervals are shown for disease-free (*DFS*), disease-specific (*DSS*) and overall survival (*OS*) for sub-groups of (**A**) estrogen receptor (*ER*)-positive and (B) human epidermal growth factor receptor (*HER2*)-positive breast cancer. The cut-off of top and bottom quartile of *NR3C1* expression was considered as GR high and GR low in the respective subtypes. Log-rank test was used to compare the survival between GR high and GR low breast cancer.

3. Figure S3: Correlation between GR expression and macrophages across different immune cell composition algorithms (EPIC, TIMER, CIBERSORT, xCELL).

4. Figure S4: Hallmark gene sets with significant enrichment in GR high and low breast cancer with FDR < 0.25.

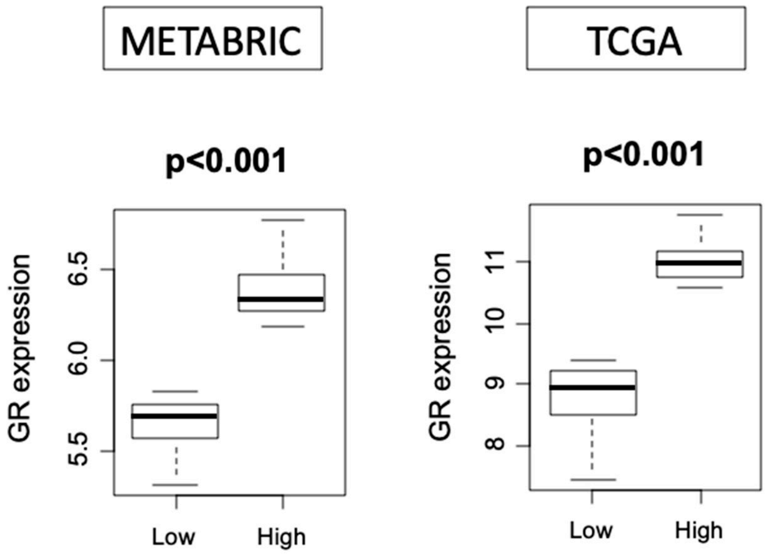

Figure S1: Highest and lowest quartiles (25%) of *NR3C1* RNA-seq expression as a cutoff to identify "High" and "Low" GR (*NR3C1*) tumor expression, respectively.

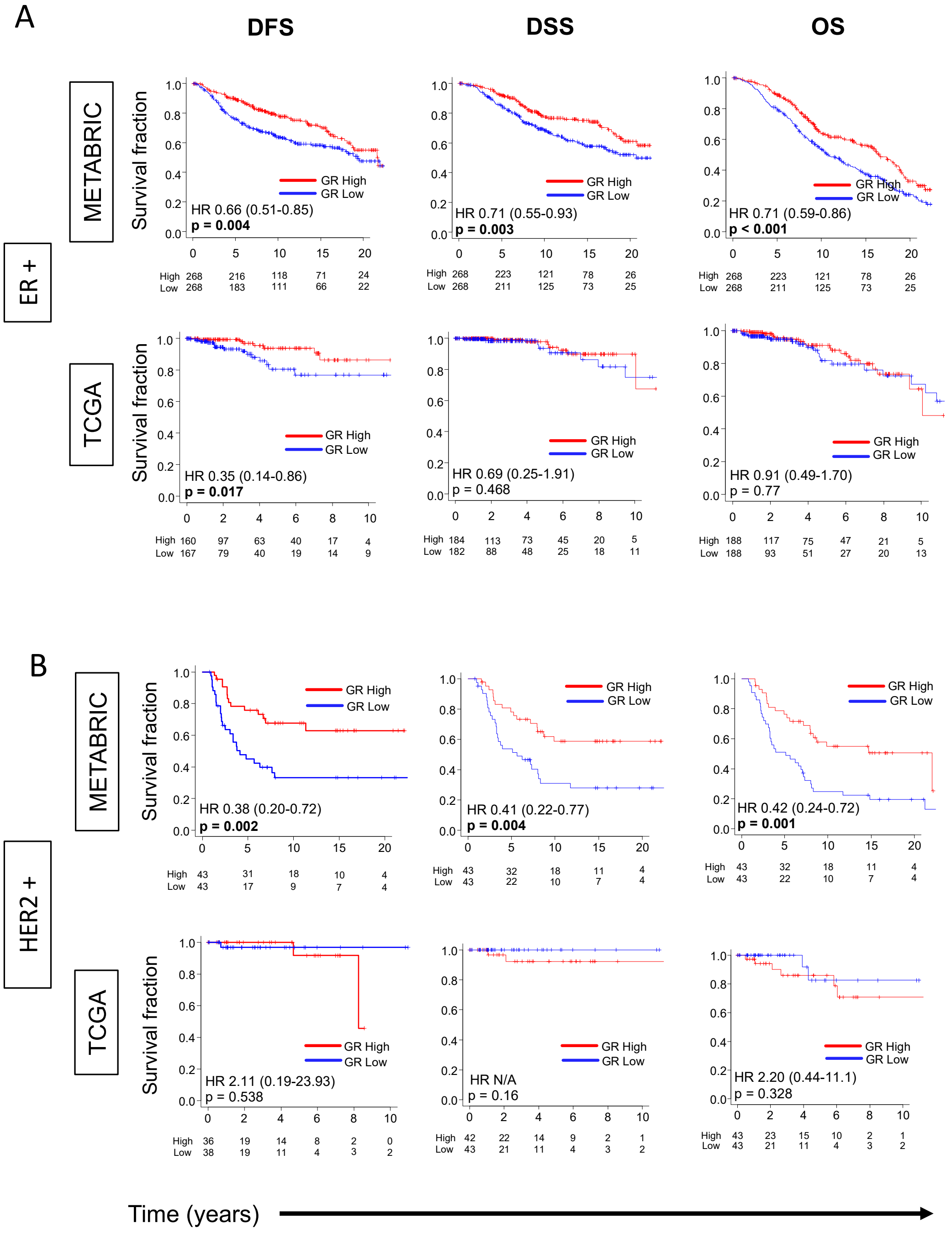

Figure S2: *GR expression and survival outcomes in breast cancer*. Kaplan-Meier survival plots comparing patients with high and low GR expression along with logrank test p values and hazard ratios (HR) with confidence intervals are shown for disease-free (*DFS*), disease-specific (*DSS*) and overall survival (*OS*) for sub-groups of (**A**) estrogen receptor (*ER*)-positive and (B) human epidermal growth factor receptor (*HER2*)-positive breast cancer. The cut-off of top and bottom quartile of *NR3C1* expression was considered as GR high and GR low in the respective subtypes. Log-rank test was used to compare the survival between GR high and GR low breast cancer.

cancer	Macrophage EPIC	Macrophage TIMER	Macrophage XCELL	Macrophage M0 CIBERSORT	Macrophage M0 CIBERSORT-ABS	Macrophage M1 CIBERSORT	Macrophage M1 CIBERSORT-ABS	Macrophage M1 QUANTISEQ	Macrophage M1 XCELL	Macrophage M2 CIBERSORT	Macrophage M2 CIBERSORT-ABS	Macrophage M2 QUANTISEQ	Macrophage M2 XCELL	Macrophage M2 TIDE	Macrophage/Monocyte MCP-COUNTER
BRCA (n=1100)	0.021	0.334	0.019	-0.19	-0.116	0.004	0.146	0.217	-0.149	0.129	0.372	0.356	0.093	-0.162	0.237
BRCA-Basal (n=191)	0.128	0.223	0.115	-0.029	0.189	0.188	0.372	0.321	0.042	0.091	0.465	0.356	0.044	-0.344	0.36
BRCA-Her2 (n=82)	0.085	0.485	0.286	-0.192	-0.028	0.032	0.204	0.23	0.132	0.215	0.372	0.115	0.176	-0.227	0.407
BRCA-LumA (n=568)	0.002	0.321	-0.008	-0.176	-0.134	0.096	0.221	0.255	-0.163	0.042	0.37	0.298	0.054	-0.227	0.321
BRCA-LumB (n=219)	0.117	0.357	0.141	-0.205	-0.133	-0.075	0.054	0.255	-0.06	0.209	0.325	0.375	0.118	-0.198	0.297

Figure S3: Correlation between GR expression and macrophages across different immune cell composition algorithms (EPIC, TIMER, CIBERSORT, xCELL).

GR High group

METABRIC

NAME	NES	FDR q-val
HALLMARK_INFLAMMATORY_RESPONSE	1.60	0.04
HALLMARK_ALLOGRAFT_REJECTION	1.55	0.04
HALLMARK_TNFA_SIGNALING_VIA_NFKB	1.55	0.04
HALLMARK_COAGULATION	1.56	0.04
HALLMARK_KRAS_SIGNALING_UP	1.60	0.05
HALLMARK_UV_RESPONSE_DN	1.56	0.05
HALLMARK_APOPTOSIS	1.51	0.06
HALLMARK_IL6_JAK_STAT3_SIGNALING	1.61	0.07
HALLMARK_IL2_STAT5_SIGNALING	1.47	0.08
HALLMARK_ANGIOGENESIS	1.43	0.10
HALLMARK_EPITHELIAL_MESENCHYMAL_TRANSITION	1.41	0.10
HALLMARK_INTERFERON_GAMMA_RESPONSE	1.41	0.10
HALLMARK_ADIPOGENESIS	1.43	0.11
HALLMARK_TGF_BETA_SIGNALING	1.41	0.11
HALLMARK_COMPLEMENT	1.61	0.12
HALLMARK_P53_PATHWAY	1.36	0.13
HALLMARK_XENOBIOTIC_METABOLISM	1.37	0.13
HALLMARK_APICAL_SURFACE	1.34	0.14
HALLMARK_BILE_ACID_METABOLISM	1.34	0.14
HALLMARK_MYOGENESIS	1.30	0.16
HALLMARK_HYPOXIA	1.31	0.16
HALLMARK_FATTY_ACID_METABOLISM	1.26	0.20
HALLMARK_APICAL_JUNCTION	1.25	0.20

TCGA

NAME	NES	FDR q-val
HALLMARK_TGF_BETA_SIGNALING	1.85	0.13
HALLMARK_KRAS_SIGNALING_UP	1.73	0.18
HALLMARK_EPITHELIAL_MESENCHYMAL_TRANSITION	1.53	0.21
HALLMARK_ANDROGEN_RESPONSE	1.74	0.22
HALLMARK_IL2_STAT5_SIGNALING	1.55	0.23
HALLMARK_HEDGEHOG_SIGNALING	1.53	0.23
HALLMARK_COMPLEMENT	1.56	0.24
HALLMARK_UV_RESPONSE_DN	1.86	0.25
HALLMARK_APOPTOSIS	1.42	0.25

GR Low group

METABRIC

NAME	NES	FDR q-val
HALLMARK_MYC_TARGETS_V2	-1.71	0.01
HALLMARK_MITOTIC_SPINDLE	-1.49	0.14
HALLMARK_G2M_CHECKPOINT	-1.44	0.15
HALLMARK_E2F_TARGETS	-1.37	0.17
HALLMARK_SPERMATOGENESIS	-1.37	0.21

TCGA

NAME	NES	FDR q-val
HALLMARK_DNA_REPAIR	-2.10	0.01
HALLMARK_MYC_TARGETS_V2	-1.20	0.01
HALLMARK_MYC_TARGETS_V1	-1.91	0.01
HALLMARK_OXIDATIVE_PHOSPHORYLATION	-1.87	0.01
HALLMARK_E2F_TARGETS	-1.60	0.09
HALLMARK_UNFOLDED_PROTEIN_RESPONSE	-1.42	0.19
HALLMARK_G2M_CHECKPOINT	-1.33	0.24

Figure S4: Hallmark gene sets with significant enrichment in GR high and low breast cancer.
